# Editorial: Brain Oscillations and Predictive Coding in the Context of Different Conscious States and Sleep-Wake Cycle: Implications for Decision Making and Psychopathology

**DOI:** 10.3389/fpsyg.2016.01768

**Published:** 2016-11-11

**Authors:** Roumen Kirov

**Affiliations:** Cognitive Psychophysiology, Institute of Neurobiology, Bulgarian Academy of SciencesSofia, Bulgaria

**Keywords:** brain oscillations, predictive coding, REM sleep, dreaming, cognition, psychopathology, evolution

Predictive coding (PC) is a neurocognitive concept, according to which the brain does not process the whole qualia of external information, but only residual mismatches occurring between incoming information and an individual, inner model of the world, thus minimizing the free energy (FE) or brain entropy. The original concept comes from the Helmholtz's view of Bayesian brain or unconscious inference (Helmholz, 1878/1971), and has been elaborated in the last decades by Karl Friston in terms of PC functioning at every level of brain anatomic and functional organization and mind-body interaction (Friston, 2010, 2012). While neurophysiologic evidence is increasingly accumulated in support of the PC concept (Friston, 2012), compelling issues remain open. (1) Is PC an evolutionary pathway which we still underscore? (2) Which neurophysiologic mechanisms support the formation, maintenance and consolidation of the inner model determining PC? (3) Whether and how these may be referenced to the “self” in normal and pathological conditions, thus representing an imperative limit of our free will? (4) Is PC continuously modulated by external environmental or internal mental information? This Research Topic features contributions from several leading scholars addressing these issues.

It is hardly a coincidence that most contributions deal with the neurophysiology of sleep and its dreaming conscious states. Increasing evidence has been provided for the active role of sleep in processes such as metabolic functions and energy balance, synaptic plasticity and memory processing, emotional regulation, and prophylactic cellular maintenance, all of utmost importance for the successful adaptation (Vyazovskiy, 2015). However, a role of sleep for PC is still generally overlooked. Particularly, the bizarre dreaming consciousness upon lack of external input during rapid eye movement (REM) sleep may provide a unique condition for generating the inner model of the world. This may be achieved by incorporating newly encoded memories into consolidated residuals of hypotheses, emotions, basic needs, and individual genetic traits. Such a continuous update of the vital context is thought to determine PC and successful adaptation during wakefulness (Hobson and Friston, 2012; Horne, 2013; Kirov, 2013).

We begin with a scholarly and authoritative hypothesis article by Hobson et al. arguing convincingly that REM sleep and its characteristics, brain oscillations, rapid eye movements and pontine-geniculate-occipital waves, neuromodulation mode, and bizarre dreaming experience, all can support a more complex function of REM sleep than proposed so far. Especially, along with the REM sleep neurobiology, the dreaming conscious state during REM is considered a virtual reality model of surroundings which could determine or modulate the PC. This opening paper also addresses evolutionary and ontogenetic meanings of REM sleep and its key role for complex relationships between primary and secondary consciousness for the successful adaptation. Next, considering the bizarre and hyperassociative nature of REM sleep dreaming, several essentially important implications for REM sleep's neurophysiology and psychology merit a particular attention. According to an insightful model of Llewellyn, REM sleep dreaming generates a prospective code which may identify a personally salient, non-obvious probabilistic pattern in past events that if mobilized into a sensorimotor image as predictive code in wake, could support cognition in wake through sensory input with appropriate action. Notably, the form taken by this prospective code may also relate to ecological contexts for the predictive brain, where REM dreaming evolved to engender-based code which associates temporally discontiguous events in evolutionary terms. The original pilot study by Scarpelli et al. demonstrates that theta electroencephalogram (EEG) oscillations during REM sleep predict successful dream recall in a state-dependent fashion, depending on current psychological states. Along with the impressive review article of Scarpelli et al., this study opens new insights about the role of dreaming for feasible associations between previous experiences and broader biological imperatives that may determine the PC.

From the view point of REM sleep's role for psychopathology, the following papers inspire fresh ideas. First, reconciling REM sleep neurophysiology, Castelnovo et al. bring advantageous perspectives for understanding REM sleep dreaming as a virtual reality model for deviant PC in schizophrenic patients. Second, the search activity concept of REM sleep dreaming proposed by Rotenberg questions adequately and timely possible disadvantages of mental imagery like lucid dreaming (LD). In particular, while LD alters normal brain activation (Voss et al., 2009), its benefits for the natural functions of REM sleep remain unclear. Third, we welcome particularly the hypothesis article of Hopkins which builds on the links between predictive coding and dreaming on one side, and psychopathology and psychoanalytic approaches on the other side. The theoretical and empirical grounds of this important contribution to the topic deserve further attention of expert readers and experimenters.

With regard to the impressive amount of data about brain oscillations and PC during wakefulness, the opinion of Sauseng et al. is an excellent emphasis on cross-frequency EEG coupling as feasible mechanisms for PC in visual search. However, it appears very important to link sleep and wake data regarding the neurophysiology of cognitive and psychopathological aspects of the PC. Thankfully, this gap is targeted by the review of Başar and Düzgün pointing to the role of slow brain oscillations for PC, consciousness, memory, behavior, and psychopathology during sleep and wakefulness.

What we learned from the valuable contributions in this research topic can be summarized as follows: (1) The undermining neurophysiologic and psychological mechanisms of PC are strongly associated with sleep and dreaming features, and particularly with those signifying REM sleep. This in turn can reveal a novel function of sleep which may be of essential importance for the successful adaptation. (2) PC appears as a universal evolutionary pathway which role for cognition and psychopathology we still underscore. What we should learn is whether PC is a genetically determined imperative which limits our free will. Addressing this question mandates further theoretical and experimental developments.

I am personally quite happy with the appearance of such contributions touching on delicate and immensely important neurophysiologic, psychological and psychiatric problems related to veritable philosophical and psychosocial issues, and having the potential to contribute to a wise management of our evolution.

## Author contributions

RK drafted the manuscript and is accountable for all aspects of the work.

### Conflict of interest statement

The author declares that the research was conducted in the absence of any commercial or financial relationships that could be construed as a potential conflict of interest.
